# Magnetoviscosity of a Magnetic Fluid Based on Barium Hexaferrite Nanoplates

**DOI:** 10.3390/ma14081870

**Published:** 2021-04-09

**Authors:** Dmitry Borin, Robert Müller, Stefan Odenbach

**Affiliations:** 1 Institute of Mechatronic Engineering, Technische Universität Dresden, 01062 Dresden, Germany; stefan.odenbach@tu-dresden.de; 2 Leibniz Institute of Photonic Technology, 07745 Jena, Germany; robert.mueller@leibniz-ipht.de

**Keywords:** ferrofluid, magnetic fluid, rheology, barium hexaferrite, nanoplates

## Abstract

This paper presents the results of an experimental study of the influence of an external magnetic field on the shear flow behaviour of a magnetic fluid based on barium hexaferrite nanoplates. With the use of rheometry, the magnetoviscosity and field-dependent yield-stress in the fluid are evaluated. The observed fluid behaviour is compared to that of ferrofluids with magnetic nanoparticles having high dipole interaction. The results obtained supplement the so-far poorly studied topic of the influence of magnetic nanoparticles’ shape on magnetoviscous effects. It is concluded that the parameter determining the observed magnetoviscous effects in the fluid under study is the ratio $V^{2}/l^{3}$, where *V* is the volume of the nanoparticle and *l* is the size of the nanoparticle in the direction corresponding to its orientation in the externally applied magnetic field.

## 1. Introduction

Conventional magnetic fluids (MFs), also well known as ferrofluids, are stable, nonconcentrated colloids based on magnetic nanoparticles. Depending on the size and concentration of the particles as well as the type of carrier medium, the rheological behaviour of MFs can be Newtonian or non-Newtonian, i.e., nonlinear depending on the shear rate, regardless of whether an external magnetic field is applied. In terms of the magnetoviscous effect (MVE), the decisive role is played by the magnetic size of nanoparticles, which determines the ability of particles to structure in an external magnetic field [1]. The structures of nanoparticles formed in the magnetic field, as a rule being chainlike aggregates, are the main cause of strong dependence on the applied field changes in the viscosity of MF. This topic has been intensively explored in the past from both experimental and theoretical viewpoints. Recent reviews on this subject have been presented, e.g., in [2,3,4]. Various technical and biomedical applications of ferrofluids have been considered in [5,6,7,8].

The studies of MF rheology mainly focus on factors such as the magnetic field, shear rate, particle volume concentration, and presence of various additives. On the other hand, the rheological properties of colloidal suspensions are known to depend on the shape of the disperse phase [9]. In the case of MF, the shape of the particles can obviously affect the structuring processes in the magnetic field as well as the orientation and destruction of particle structures as a result of the shear flow of the fluid. Rather few scientific publications on the subject are known [10,11,12]. It is related to the fact that ferrofluids, which can be commercially purchased or obtained relatively easily for use in a laboratory environment in quantities sufficient for rheological measurements, are usually based on spherical-shaped magnetic nanoparticles. In [10] the behaviour of a MF containing cobalt nanodiscs was studied and compared to those of a MF based on spherical cobalt nanoparticles. In addition, [11] examined a ferrofluid based on cobalt nanofibres. There are also known research publications on ferrofluids based on barium hexaferrite nanoparticles with a platelike shape. In [13], a MF based on barium hexaferrite nanoparticles having a spherical shape as well as an aspect ratio of up to ∼2 was introduced for the first time. At room temperature, these nanoparticles are super-paramagnetic, however, at low *T*, they become ferrimagnetic because of the magnetocrystalline anisotropy, which is higher than that of iron oxide. The study [13] presents the results of measurements of magnetic properties as well as the viscosity obtained using a rotational viscometer equipped with a combined Searle- and Couette-type concentric cylinder double gap geometry are presented for a MF based on these particles. The authors reported a significant MVE, i.e., an increase in the viscosity of a fluid in an external magnetic field. At the same time, this study does not provide information on the relationship between fluid viscosity and shear rate. The field-dependent flow behaviour of a ferrofluid based on barium ferrite platelets suspended in paraffin and barium-strontium hexaferrite suspended in mineral oil is briefly discussed in [12] and in [14], correspondingly. The authors reported the power law behaviour of these fluids, but the used measurement technique raises questions in regard to the strict physical interpretation of the obtained results. The commercial device used in measurements is designed to evaluate the performance of magnetorheological fluids, i.e., highly concentrated suspensions of magnetic microparticles, in terms of the fluids’ effective application in damping and shock-absorbing devices. Thus, an issue of the influence of a magnetic field and shear flow on the MVE in barium hexaferrite MFs is definitely not sufficiently studied. Although, magnetic and structural properties of such ferrofluids, as well as various methodological issues of synthesis of corresponding nanoplates and their physical and chemical properties have been studied in detail, for example, in [15,16,17,18,19,20,21,22], therefore, they are not a priority objective of this study.

This work focuses on the evaluation of magnetoviscosity and yield-stress behaviour in a ferrofluid based on barium hexaferrite nanoplates dispersed in mineral oil.

## 2. Materials and Methods

### 2.1. Nanoparticles and Magnetic Fluid

M-Type barium hexaferrite ${\mathrm{BaFe}}_{12-2x}{\mathrm{Me}}^{2+}{\;}_{x}{\mathrm{Me}}^{4+}{\;}_{x}O_{19}$ nanoparticles were prepared by a glass crystallisation method [15,18]. In this method, particles were formed during a temperature treatment leading to crystallisation in a glass matrix, which was subsequently dissolved. With this method, oxide powders in good phase purity can be prepared. Due to their isolated growth in a solid matrix, the particles are single crystalline. The intrinsic magnetic properties are adjusted by substitution elements that can be added homogeneously in the state of the melt.

The glasses were prepared from suitable components of the corresponding ferrite (initial materials: ${\mathrm{BaCO}}_{3}$ and $\alpha -{\mathrm{Fe}}_{2}O_{3}$) and a glass network former ($B_{2}O_{3}$) by a rapid cooling (about $10^{4}$ K/s) that should prevent nucleation and particle growth. A temperature treatment of the glass between the glass transformation and softening temperature caused a phase separation and crystallisation of the particles occurred. To obtain the particles, the matrix was dissolved by acetic acid. The acidic slurry was washed and dried.

Substituting Fe by Co–Ti leads to a decreasing magnetocrystalline anisotropy, as reported in [16]. At a substitution degree of x∼1.0, this results in the strong decrease of the coercivity and the relative remanence, which is a ratio of remanent magnetisation $M_{r}$ to saturation magnetisation $M_{s}$; this is caused by the transition from uniaxial to planar magnetocrystalline anisotropy. Substituting Fe with Co–Ti also leads to smaller particle sizes.

**Figure 1:**
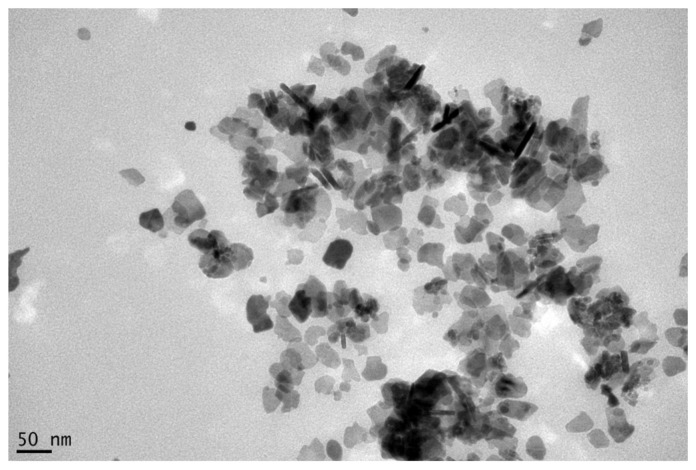
TEM image of the prepared barium hexaferrite nanoplates (TEM is performed by Katrin Buder, Leibniz-FLI, Jena, Germany).

For the preparation of our sample glass, flakes from the system 40BaO-33-B_2_O_3_-27(Fe_2_O_3_+CoOTiO_2_) (mol%) with a substitution degree of x∼1.1 were heat-treated at 650 ${}^{{}^{\circ}}$C for 4 h. The effect of decreasing magnetocrystalline anisotropy by CoTi-substitution might be superimposed by super-paramagnetism due to the small particle size. The magnetic nanoparticles we obtained using this method are platelike with an average size of ∼35 nm by ∼4 nm (thickness). A TEM image (Zeiss DSM 960, Jena, Germany) of the particles is given in Figure 1. Magnetic measurements were performed using a vibrating sample magnetometer Lake Shore 7407 (Westerville, OH, USA). The particles in powder form have the following magnetic properties: coercivity $H_{c}$ = 7.6 kA/m; relative remanence $M_{r}/M_{s}$ = 0.170; specific saturation magnetisation σ = 37.8 Am${}^{2}$/kg.

To obtain a ferrofluid, the prepared barium hexaferrite nanoparticles were dispersed in a mineral oil P3 (Pfeiffer Vacuum Technology AG, Asslar, Germany; kinematic viscosity ν = 312 mm${}^{2}$/s and density ρ∼870 kg/m${}^{3}$ at 20 ${}^{{}^{\circ}}$C) using oleic acid as a surfactant. Measured specific magnetisation of the fluid is σ = 9.0 Am${}^{2}$/kg, which corresponds to the particle concentration of ca. 23.8 wt%, i.e., ca. 4.7 vol%.

### 2.2. Rheometric Measurements

**Figure 2:**
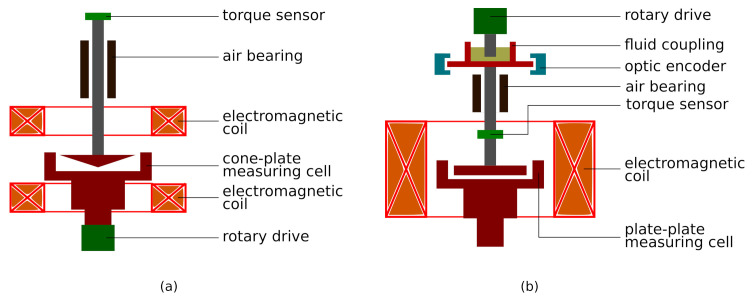
Rheometric equipment: (**a**) shear rate and (**b**) shear-stress-controlled experimental setup. A detailed description and evaluation of the equipment is given in [1,23], respectively.

An experimental evaluation of the influence of magnetic field and shear rate on the MVE of the MF under consideration was performed using a special shear-rate-controlled rheometer (Figure 2a). The rheometer (in-house engineering) was developed in the past specifically for ferrofluids and is described in detail in [1]. The cone-plate made of brass measuring geometry is combined with a Couette region and uses a cone with a 3${}^{{}^{\circ}}$ open angle and diameter of 76 mm. In the experiments on MVE, the bottom plate of the measuring cell rotates while the torque *T* transmitted by the fluid is measured by a sensor on a cone suspended in the air bearing. A homogeneous externally applied magnetic field is perpendicular to the shear flow and is provided by a pair of magnetic coils in the Helmholtz configuration. The conducted measurements used shear rates $\gamma^{\prime }$ in the range of 1–10 s${}^{-1}$ and the magnetic field *H* in the range of 0–30 kA/m. An experimental sample of the ferrofluid was presheared after each magnetic field application to ensure its homogeneous microstructural state through mechanical disintegration of the possible residual particle structures. The quantitative MVE assessment, denoted as *R*, is performed in accordance with [1] as

(1) $$R\left(\gamma^{\prime }\right)=\frac{T_{H}-T_{H=0}}{T_{H=0}}$$

where $T_{H=0}$ is the torque obtained for the zero magnetic field and $T_{H}$ is the torque obtained for the field with a strength *H*.

To evaluate yield stress, the shear-stress-controlled experimental setup reported in [23] was used (Figure 2b). The measuring geometry of the system is a plate–plate configuration with an upper plate diameter of 76 mm. The material of this measuring geometry is aluminium. A homogeneous magnetic field is perpendicular to the direction of the applied deformation and is provided by a large electromagnetic coil around the measuring cell. The upper plate is suspended in the air bearing, while the bottom plate is statically fixed. The measuring gap is varied in the range of 0.25–1.25 mm. In evaluating yield stress, the increasing shear stress is applied to the fluid sample at rest until the first continuous measuring motion, as determined by the optical encoder. The maximum stress at which no motion is detected is interpreted as the yield stress $\tau_{y}$. The dependence $\tau_{y}\left(H\right)$ is obtained by repeating the procedure at various *H* up to 60 kA/m, in doing so, the fluid sample is presheared after each magnetic field application.

All results reported below were obtained at 20 ${}^{{}^{\circ}}$C.

## 3. Results and Discussion

### 3.1. Magnetoviscous Effect

**Figure 3:**
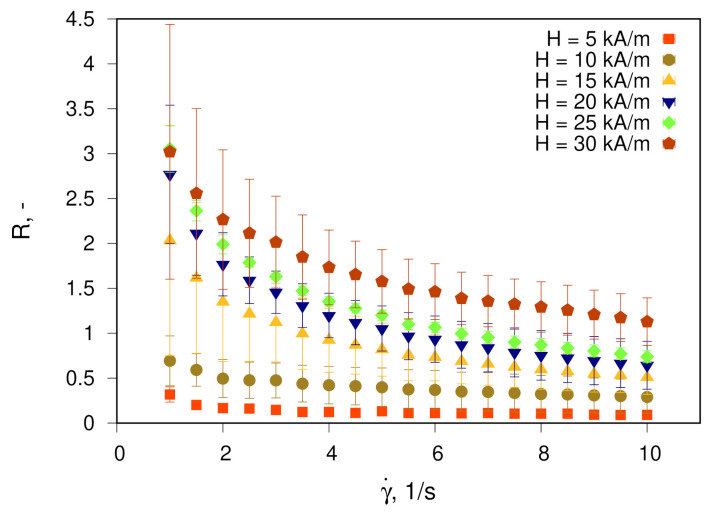
Shear dependence of the magnetoviscous effect in studied fluid for various *H*.

**Figure 4:**
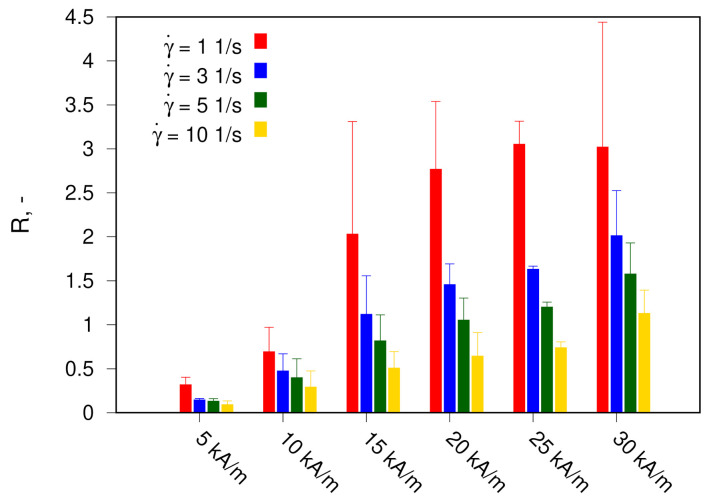
The magnetoviscous effect in studied fluid for 4 different shear rates at various *H*.

The results of the MVE measurement are shown in Figure 3 and Figure 4. Figure 3 demonstrates the dependence of parameter *R* on the shear rate $\gamma^{\prime }$ for various magnetic fields *H*, while in Figure 4, values of *R* for four different shear rates at various *H* are given as a bar chart.

The higher the magnetic field strength and the smaller the shear rate, the higher the MVE. Qualitatively observed dependencies are typical for ferrofluids with a pronounced effect of the formation of nanoparticle structures in a magnetic field—in particular, chainlike or droplike structures. These particle aggregates are oriented along the applied magnetic field on the one hand, while on the other hand, shear flow deflects the aggregates from this direction or separates them into smaller structures or individual particles. As a result, the MVE is a product of the competition between magnetic field strength and shear flow both qualitatively and quantitatively [4]. As a criterion for evaluating the ability of particles to form structures under the influence of a magnetic field, the so-called interaction parameter is used [24]. The interaction parameter is the ratio of dipole interaction energy of two particles in contact to their thermal energy and is calculated as

(2) $$\lambda =\frac{\mu_{0}{M_{0}}^{2}V^{2}}{4\pi l^{3}k_{B}T}$$

where $\mu_{0}$ is the vacuum permeability, $M_{0}$ is the spontaneous magnetisation of the particle magnetic material, *V* is the volume of the nanoparticle, $k_{B}$ is the Boltzmann constant, *T* is the temperature, and *l* is the characteristic distance between two particles. In the case of spherical nanoparticles, the parameter *l* is their diameter. For nanoparticles to be able to form structures in a magnetic field, the energy of their dipole interaction must not be less than the thermal energy, i.e., the following condition must be met:

(3) λ≥1

For a more accurate assessment, the thickness of the coating layer on the surface of nanoparticles should be taken into account [1]. In the case of spherical nanoparticles, a modified parameter is estimated as

(4) $$\lambda^{*}=\lambda {\left(\frac{d}{d+2s}\right)}^{3}$$

where *s* denotes the coating thickness, which is usually of several nanometres and *d* is the particle diameter. Moreover, since ferrofluids are usually polydispersed, it may make sense to consider the weighted interaction parameter taking into account the particle size distribution by classes. Nevertheless, when particles size distribution and parameters of the coating are not exactly known, Equations (2) and (3) make it possible to roughly estimate the critical characteristic size of nanoparticles at which structuring will take place. For the ferrofluid under study, the parameter λ is significantly higher than unity when the thickness of nanoplates is taken as the characteristic distance *l*. It can be assumed that barium hexaferrite nanoparticles are structured in a field with flat surfaces facing each other. In the opposite case, i.e., orientation of platelet particles by the plane along the field, the parameter λ<1 and one would not expect a high MVE. Moreover, quantitatively, the MVE of the barium hexaferrite ferrofluid is comparable to the effect obtained under similar experimental conditions for fluids based on nanoparticles exhibiting high dipole interaction. This can be seen in particular in Table 1, which provides an overview of the parameters of some ferrofluids with an indication of the maximum experimentally measured MVE. On the other hand, the sample under test is not emitted by any specific behaviour and one could conclude that the shape of the nanoparticles does not play a significant role in terms of changes in the fluid magnetoviscosity. However, it should be noted that the spontaneous magnetisation of barium hexaferrite is much smaller than that for cobalt and magnetite, and the effect reaches the same values in comparable magnetic fields and at comparable shear rates. The reason for this is precisely related to the shape of the nanoparticles, or rather to their orientation when structuring in an external magnetic field. It is obvious that for spherical particles, the volume *V* is the determining factor of dipole interaction energy (see Equation (2)), while for nonspherical particles, it is the ratio of the square of their volume to the characteristic size in a cube, i.e., $V^{2}/l^{3}$, where *l* represents the distance between the centres of the two particles in contact. In other words, in order to obtain high λ values, one needs as large a nanoparticle volume *V* as possible and the characteristic size *l* as small as possible at the same time. In the case of flat nanoparticles of barium hexaferrite, which are oriented with a plane perpendicular to the external magnetic field, the lower values of spontaneous magnetisation $M_{0}$ compared to cobalt and magnetite are compensated by the corresponding $V^{2}/l^{3}$ ratio. In this regard, it would be interesting to investigate in the future the effect of the aspect ratio of nanoplates on magnetoviscosity. Giant effects, i.e., significantly higher than previously reported, can be expected from concentrated ferrofluids based on very thin nanoflakes, if only the $V^{2}/l^{3}$ ratio is taken into account. It should be noted, however, that the stability of chains of nanoparticles of different shapes and sizes may also be different in shear flow. For example, nanoplates, depending on the overlap area, could slide against each other more easily than spherical particles. This will have the consequence of reducing the MVE.

### 3.2. Yield Stress

**Figure 5:**
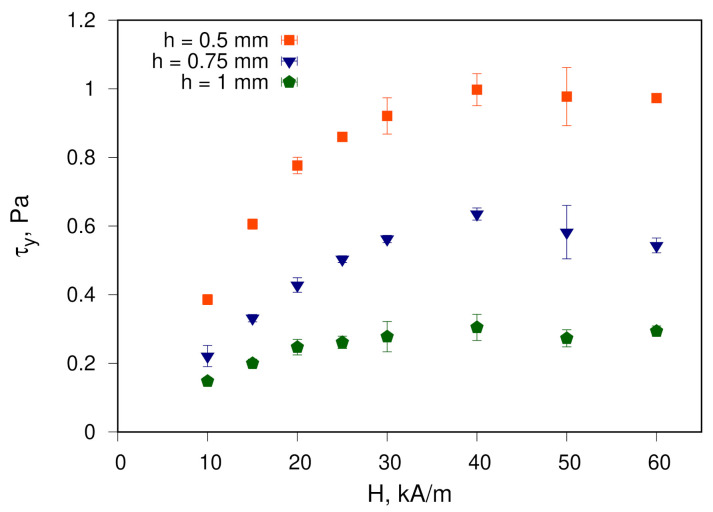
Yield stress $\tau_{y}$ for the studied ferrofluid as a function of the applied magnetic field for various distances between the walls in plate–plate geometry.

**Figure 6:**
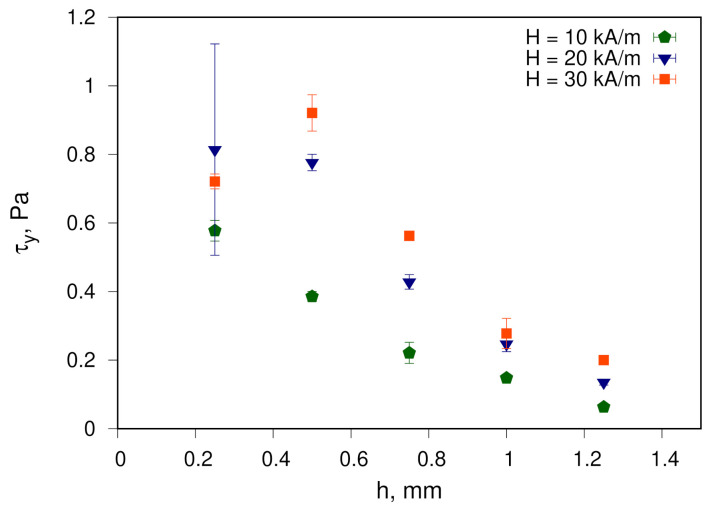
Yield stress $\tau_{y}$ for the studied ferrofluid as a function of gap distance for different magnetic field strengths.

The results of yield-stress evaluation as a function of the magnetic field strength and the size of the gap between the plates of the measuring geometry are shown in Figure 5 and Figure 6, respectively.

As the magnetic field strength increases, the value of the measured yield stress $\tau_{y}$ increases when reaching the plateau in fields larger than 30 kA/m. Increasing the gap of the measuring geometry reduces the yield-stress values.

The obtained values are comparable to those for fluids based on cobalt nanoparticles, which are characterised by a high dipole interaction energy. For example, for fluids #3 and #4 from Table 1, the yield-stress values are in the range of 0.045–3 Pa. Nevertheless, an objective quantitative comparison with results obtained for other ferrofluids—e.g., those considered above regarding MVE—is not meaningful. The reason for this is different conditions of measurements, primarily, the specification of used measuring geometry, e.g., the gap size. Moreover, for ferrofluids studied outside our scientific group, yield-stress values are either obtained from flow curves fitting rather than from experiments, or are not reported at all. On the other hand, it is possible to compare the evaluated yield-stress dependencies on magnetic field strength at different values of the gap between the plates of measuring cell with the results obtained for a ferrofluid sample with spherical cobalt particles, as reported in [31]. In the context of yield stress, the behaviour of fluids observed in [31] and in the current study is qualitatively similar. Quantitative values are also in the same order of magnitude and are slightly larger for barium hexaferrite nanoplates. It can be referred to the higher concentration of magnetic material in addition to the discussion given above regarding dipole interaction energy (Section 3.1).

Qualitatively, observed behaviour also correlates with the model of formation and destruction of particles aggregates, which could be chain- or droplike ones [4]. In particular, this behaviour corresponds with the mechanism for breaking down the nanoparticles domain overlapping the walls of the measuring cell [32]. Quantitatively, the values obtained experimentally for various ferrofluids are significantly lower than those predicted by the model considered in [33]. Thus, the question of the physical mechanism responsible for yield-stress behaviour remains open. Moreover, the experiments carried out so far primarily still do not allow conclusions to be made regarding the interaction of nanoparticle aggregates with the surface of the measuring geometry. Furthermore, no global conclusion can be drawn about the influence of the geometric shape of nanoparticles on the yield stress in ferrofluids. The global evaluation requires consideration of MFs with nanoplates having various aspect ratios.

### 3.3. Concluding Remarks

As a final conclusion, it should be emphasised once more that the energy of dipole interaction of magnetic nanoparticles is to be accounted as the most important factor of the magnetoviscous behaviour of MFs. In the context of the ferrofluid considered, the characteristic size of the nanoparticles is determinant, i.e., the nanoparticle orientation in the field during structuring regardless of their geometrical shape. The shape of the particle aggregates is independent of the shape of individual nanoparticles. On the other hand, the particle shape can be important in the context of aggregates interaction with the measuring geometries. Moreover, the stability of chains of nanoparticles of different shapes and sizes may also be different in a shear flow. Thus, no generalised statement can be made about the influence of the geometric shape of nanoparticles on the MVEs in ferrofluids.

Further researches are required to fully understand the physical processes underlying the rheological properties of ferrofluids based on nanoparticles with high dipole energy. In particular, targeted microstructural evaluations including numerical simulations could clarify this issue. The development of a concentrated ferrofluid based on thin nanoflakes is also seen as promising, for which gigantic MVEs can be expected. Another experimental question that has not been sufficiently addressed is the comparative analysis of the rheological behaviour of MFs of completely different compositions at different ranges of shear rates. In this context, comprehensive studies of various MFs under completely identical experimental conditions are required.

From a practical point of view, the results presented can serve as the basis for selecting a ferrofluid for a particular type of application; particularly, when a fluid with a high MVE is required. However, it should be noted in this regard that the study is limited to a narrow range of shear rates. Therefore, further, more in-depth studies would be required to assess the potential uses of the investigated ferrofluid.

## Figures and Tables

**Table 1:** Overview of certain parameters and the magnetoviscous effect (MVE) of various types of nanoparticles based magnetic fluids: samples based on particles with high dipole interaction are regarded.

#	Carrier Liquid	Nanoparticles			max.	$\gamma^{\prime }$, 1/s	H, kA/m	max.	Ref.
		Type	Shape	Size	ϕ, %			MVE	
1	synthetic ester	$Fe_{3}O_{4}$	spheres	∼13 nm	6.3 vol.	0.5	5–50	∼2	[25]
2	polyethylsiloxane	$Fe_{3}O_{4}$	spheres	∼7.5 nm	3.6 vol.	100	10–100	∼0.2–2.2	[26]
3	silicon oil DC 702	Co-based	spheres	∼8 nm	2.8 vol.	0.3	5–30	∼2–30	[27]
4	n-cetane	Co-based	plates	5 × 20 nm	0.1 vol.	∼2.5	5	∼20	[10,28]
5	mineral oil P3	$\gamma -Fe_{3}O_{4}$	clusters	50–70 nm	0.1 vol.	0.2–2	10–30	∼0.6–60	[29]
6	paraffin	Ni	clusters	175–350 nm	40 wt.	5	5–90	∼0.2–9.5	[30]
7	mineral oil P3	BaFe-based	plates	4 × 35 nm	4.7 vol.	1–10	5-30	∼0.3–3	this study

## Data Availability

The data presented in this study are available on request from the corresponding author.

## Abbreviations

The following abbreviations are used in this manuscript:

MF	Magnetic fluid
MVE	Magnetoviscous effect
